# Nutraceutical Supplements in the Management and Prevention of Osteoarthritis

**DOI:** 10.3390/ijms17122042

**Published:** 2016-12-06

**Authors:** Paola Castrogiovanni, Francesca Maria Trovato, Carla Loreto, Houda Nsir, Marta Anna Szychlinska, Giuseppe Musumeci

**Affiliations:** 1 Department of Biomedical and Biotechnological Sciences, Human Anatomy and Histology Section, School of Medicine, University of Catania, 95100 Catania, Italy; pacastro@unict.it (P.C.); carla.loreto@unict.it (C.L.); marta.sz@hotmail.it (M.A.S.); 2 Department of Clinical and Experimental Medicine, University of Catania, 95100 Catania, Italy; trovatofrancesca@gmail.com; 3 Department of Molecular and Cellular Biology and Plant Physiology, Centre of Biotechnology of Borj Cedreya, University of Carthage, Carthage 2050, Tunisia; houda.nsir@gmail.com

**Keywords:** nutraceuticals, osteoarthritis, prevention, diet

## Abstract

Nutraceuticals are dietary compounds which have a role in the balance of anabolic and catabolic signals in joints. Their regulatory function on homeostasis of cartilage metabolism nutraceuticals is increasingly considered for the management and, above all, the prevention of osteoarthritis (OA). OA is a degenerative disease characterized by cartilage and synovium inflammation that can cause joint stiffness, swelling, pain, and loss of mobility. It is a multifactorial disease and, due to the great percentage of people suffering from it and the general increase in life expectancy, OA is considered as one of the most significant causes of disability in the world. OA impairs the structural integrity of articular cartilage that greatly depends on a balance between the anabolic and catabolic processes which occur in chondrocytes and synovial fluid of the joints, therefore the integration with nutraceutical compounds in diet increases the treatment options for patients with established OA beyond traditional rehabilitation, medications, and surgical strategies. In our review, with respect to the current literature, we highlight some of many existing nutraceutical compounds that could be used as integrators in a daily diet thanks to their easy availability, such as in olive oil, fish oil, and botanical extracts used as non-pharmacologic treatment.

## 1. Introduction

Osteoarthritis (OA) is a degenerative disease characterized by cartilage and synovium inflammation that can cause joint stiffness, swelling, pain, and loss of mobility [1,2,3]. OA is a very complex and multifactorial disease. Due to the high percentage of people suffering from this disease, with a greater percentage in women after menopause (18%) than in men (9.6%), and the increase in life expectancy, OA is considered one of the most significant causes of disability in the world [4,5,6,7]. Although OA mainly affects the joints of knees, hands, and hips [8], it also results in alterations in other joint tissues such as ligaments, synovium and subchondral bone [9]. OA impairs the structural integrity of articular cartilage that greatly depends on a balance between anabolic and catabolic processes, which occur in chondrocytes and synovial fluid of the joints [10,11]. Nutraceuticals are dietary compounds that, from data in literature, seem to play a role in these processes within articular cartilage [12]. Although there are many papers in the scientific literature concerning the use of a great variety of nutraceuticals as an alternative treatment of OA, the aim of our descriptive review is to highlight the importance of non-invasive strategies in the treatment of OA through the use of the most common and easily available nutraceuticals, such as olive oil, fish oil, and botanical extracts. The integration with nutraceutical compounds in diet increases the treatment options for patients with established OA beyond the traditional rehabilitation, medications, and surgical strategies such as non-pharmacologic treatment [13].

## 2. Micro and Macroscopic Features in Early and Severe Osteoarthritis (OA)

**Figure 1:**
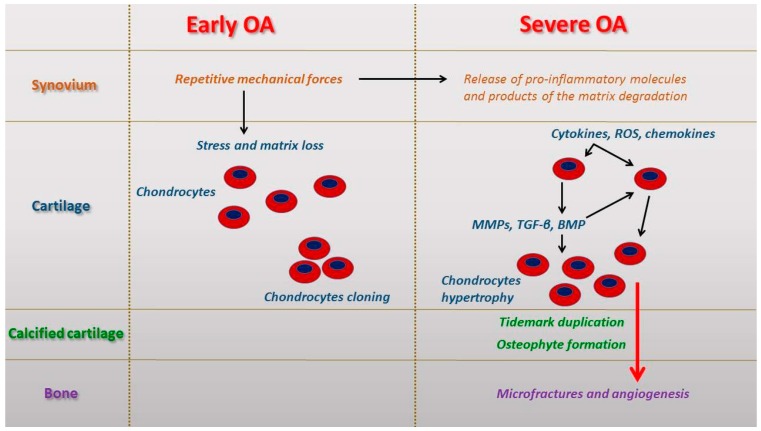
Early and severe osteoarthritis (OA) causes and molecular events in the different tissues involved in the joints. BMP: Bone morphogenetic proteins; MMPs: Matrix metalloproteinases; ROS: Reactive oxygen species; TGF-β: Transforming growth factor β.

OA is characterized by impairments in the structure and functionality of joint cartilage in consequence of an imbalance between anabolic and catabolic processes in the cartilage tissue that could cause its degradation; if cartilage degradation exceeds reparative processes, the OA goes on and advances [14,15] (Figure 1). This degenerative disease is characterized by several changes (narrowed joint space, thickening, formation of osteophytes, and cysts in the subchondral bone) that are radiographically visible, even if radiographs do not indicate the degree of cartilage degeneration [16]. It is possible to detect osteophytes also through magnetic resonance imaging (MRI), that allow us also to detect geodes or subchondral cysts in advanced stages of OA [17]. Microscopic alterations in joint cartilage are evaluated by the Mankin [18] score or a modified version by Sakakibara et al. [19], that consider several factors such as cell morphology, extracellular matrix staining, and appearance of the tidemark. The highest scores highlighting the most severe damage of joint tissues are 14 for the Mankin score and 32 for the modified Mankin score. Alterations to healthy joint cartilage usually do not exceed grades of 1–3 [20]. Histological grading criteria of Kraus′ modified Mankin score [18,21] and histopathology OARSI system [22,23] are used as semi-quantitative methods.

Cartilage is mainly composed of collagen type II and the proteoglycan aggrecan and it is characterized by viscoelastic and compressive properties thanks to the extracellular matrix [24,25,26,27]. Healthy joint cartilage has a smooth surface and it is white, shiny, and elastic. In OA, cartilage instead shows a dull and irregular surface with discoloration and softening and more synovial fluid may be produced, with newly invaded blood vessels [28]. In particular, at the early stage of degeneration, minimal changes are detected in the cartilage surface in which glycosaminoglycans remain homogenously distributed; as the disease progresses, there is a loss of proteoglycans, and in severe OA the cartilage surface is rough and broken by fissures and cracks [16].

In healthy joint cartilage, four layers are recognizable: superficial zone, middle zone, deep zone, and calcified zone. In the superficial zone, cells are flat and spindle-shaped, parallel to the joint surface [14,26]. The superficial zone contains the majority of collagen fibers, parallel to the surface, which results in high tensile modulus to resist shear stress at the joint surface [14]. In early OA, mild fibrillations are found in the superficial zone [16] and cartilage presents thickening, a consequence of hypertrophy. As the disease advances, cells of the intermediate and radial zone show mild to moderate hypercellularity; necrotic chondrocytes with pyknotic nuclei in the intermediate and radial zone are found; the synovial membrane includes hyperplasia of synovial lining cells, thickening of the synovial membrane, infiltration of inflammatory cells, and fibrosis [14,29]. In severe OA, the cartilage shows extensive degeneration: hypertrophic villi and full-thickness defect areas can be seen where the cartilage is missing completely and the subchondral bone is exposed; the subchondral plate itself is thicker and more dense; cells are arranged in clusters especially around fissures or disappear completely as the disease progresses; the cartilage is replaced by fibrocartilaginous, scar-like tissue with fibroblast-like cells [16,28]. In other cases, full-thickness defects develop, where the bone lacks the cartilage completely; the loss of proteoglycan content reaches the deep zones; the tidemark becomes unclear and finally is invaded by blood vessels from the subchondral bone, which penetrate into the calcified zone [28]. Osteophytes are found in early stages of the diseases, but become more pronounced in advanced stages of OA. The rate of OA progression depends on species and joint localization, and the extent of damage could be dependent on the joint area, which can be explained by different loading conditions in distinct regions [28].

Articular cartilage is not vascularized nor innervated, so nutrients and cellular repair molecules are transported to the chondrocytes by diffusion from the synovial fluid. Thus, articular cartilage has limited capacities for self-regeneration and, in OA, shows reduced mechanical capacities compared to healthy cartilage [30]. Chondrocytes are very active cells but they normally do not divide, so only small defects associated with minimal loss of matrix components can be repaired by regeneration; if more wide defects exceed the repair capacity, the damage can become permanent [14]. Because OA involves progressive loss of the structure and functionality of articular cartilage due to an imbalance between anabolic and catabolic processes in the cartilage tissue, preventive and therapeutic interventions are necessary to prevent OA and/or improve the regeneration capacities of joint cartilage [30].

## 3. Nutraceuticals

### 3.1. Fish Oil

The effectiveness and precise benefits of fish oil intake in patients with OA are still far from well understood. In fact, in vitro and in vivo studies showed a dose-dependent decrease in induced inflammatory destruction of cartilage tissue associated with fish oil supplementation [31]. Proposed mechanisms for the anti-inflammatory actions of n-3 fatty acids, eicosapentaenoic acid (EPA), and docosahexaenoic acid (DHA) from fish oil include competition with n-6 fatty acids; the EPA and DHA derived anti-inflammatory molecules called resolvins; the competition for receptors of n-3 products with proinflammatory molecules; the reduction in gene expression of cytokines, cyclo-oxygenase 2, and degrading proteinases; the interference in the signaling pathways of inflammation; and the reduction in lymphocyte proliferation [31]. The lack of human clinical trials showing the effects of fish oil supplementation in patients with OA is probably the most relevant hindrance to fish oil being routinely recommended [31]. Recently, a clinical trial by Hill and colleagues, demonstrating a better performance of low versus high doses of fish oil supplementation [32], provoked another heated debate on the effective utility of this compound [33].

### 3.2. GAGs (Glucosamine Sulfate, Chondroitin Sulfate, and Hyaluronic Acid)

As is well known that hyaluronic acid, glucosamine sulfate, and chondroitin sulfate are glycosaminoglycans (GAGs) synthesized by chondrocytes and synoviocytes, and are basic components of the extracellular matrix and synovial fluid. GAGs can also be introduced with food, thus there is a growing interest towards the nutritional supplementation of these molecules, to protect the joints from possible alterations caused by trauma or wear and, therefore, for OA prevention [34]. From clinical and preclinical data, supplementation of glucosamine sulfate seems to improve joint function and reduce pain and also appears to stimulate cartilage regeneration, thus inducing a regression of OA [34]. Although not all the related papers are in agreement and in many clinical trials there is a large number of subjects who did not benefit from treatment with glucosamine, an advantage of this treatment is that glucosamine has low and rare adverse effects so that it could represent a viable option for the management of OA [35]. In two randomized controlled trials, glucosamine sulfate, as well as chondroitin sulfate, mitigated the catabolic and degenerative processes thanks to their anti-inflammatory and antioxidant properties [36,37]. A pharmacokinetic study, however, suggests that the simultaneous ingestion of glucosamine and chondroitin sulfate has no synergic effect due to the competition between the two molecules in intestinal absorption [38]. Therefore, the therapeutic indication is to prevent their co-ingestion and to use different times of ingestion per day with appropriate dosages, taking into account the individual clinical cases [39]. However, other authors evidenced that glucosamine and chondroitin sulfate alone or in combination did not reduce pain in the overall examined group of patients with knee OA and that exploratory analyses suggested this kind of treatment only in a subgroup of patients with moderate-to-severe knee pain [40]. According to other authors, the optimal use of glucosmine sulfate and chondroitin sulfate to improve the status of the pain and stiffness is achieved by further integration with n-3 fatty acids eicosapentaenoic acid (EPA) and docosahexaenoic acid (DHA) from fish oil [35,41]. Additionally, hyaluronic acid (HA), another GAG, has beneficial properties on the joints. In vitro, it improves the mechanical properties of the synovial fluid and has a biochemical regulatory role on joint tissues [42]. HA is usually used in clinical contexts through local injections in the joint, with a reduction in pain and improvement in function [43]. Many authors also suggest oral use of HA that is absorbed in the intestine and released into joint tissues [44]. The results of improvement obtained with WOMAC scores are encouraging, although further research is needed to identify any long-term positive results [39].

### 3.3. Olive Oil

Olive oil is the principal fat and one of the cornerstones of the Mediterranean diet. The anti-inflammatory properties of olive oil are attributed to its phytochemicals, such as the phenolic compounds and monounsaturated fatty acids (MUFAs) [27]. In rats, it was demonstrated that an olive oil supplemented diet improves cartilage recovery after anterior cruciate ligament transection [27]. Only one double blinded, randomized clinical trial in the literature demonstrated that the topical application of olive oil improved pain and physical function in patients affected by knee osteoarthritis, confirming the utility of the traditional methods used in some rural areas of Iran [45]. Also in this case, the lack of clinical trials demonstrating the effect of olive oil supplementation with diet limits the recommendation of this compound.

### 3.4. Methionine

Methionine is an essential amino acid for humans, since the human organism is not able to synthesize it and therefore it is taken with the diet. The active form of methionine is *S*-adenosylmethionine (SAMe) is a precursor of glutathione. SAMe has antioxidant properties and, in the joints, provides levels of glutathione peroxidase, an antioxidant enzyme [46]. In addition, SAMe inhibits enzymes that degrade the cartilage protecting its proteins and proteoglycans [39]. Some researchers show that SAMe promotes anabolic processes of cartilage, thus having a regulatory function in cartilage regeneration [47]. It was also shown that, in patients with OA, treatment with SAMe has a more beneficial effect in the long term compared to treatment with nonsteroidal anti-inflammatory drugs (NSAIDs) [48], even if in some clinical trials SAMe was found to be equally effective as the NSAID and more effective than placebo for pain and function [49,50]. SAMe has proven to be an even more effective supplement in patients with OA and with liver or kidney diseases characterized by a difficulty in the metabolic activation of methionine [39]. Usually, the use of SAMe envisages a dose of 800–1600 mg per day and the indication of a sufficient intake of folate and vitamin B [39].

### 3.5. Undenatured Type II Collagen

Undenatured type II collagen (UC-II) is a nutritional supplement derived from chicken sternum cartilage [49]. Data in the literature show that the treatment of OA patients with UC-II increases the mobility and the functionality of the joints and reduces pain [49,50,51,52]. In an animal model, UC-II influences the humoral and cellular immune response through the T-regulatory cell secreting cytokines such as IL-10 and transforming growth factor-β which inhibit the immune response to collagen type II present in the extracellular matrix of the articular cartilage [53,54]. Treatment with UC-II prevents, therefore, the proinflammatory overreaction of the immune system against the articular cartilage in patients with OA [39].

### 3.6. Botanical Extracts

Botanical extracts are a large variety of substances obtained from plants and used as additives in diet.

Avocado/soy unsaponifiable (ASU) components are a sterol-rich hydrolyzed lipid fraction extract from avocado and soybean. ASU has anabolic and anti-inflammatory properties on chondrocytes, indeed in vitro ASU inhibits inflammatory cytokines such as IL-1, IL-6, IL-8, and prostaglandin E2 [55]. Moreover, ASU stimulates transforming growth factor production, collagen, and aggrecan synthesis [56]. In treatment of OA, the use of 300 mg per day of ASU determines an improvement of OA symptoms, even if further research is necessary to better understand the role of ASU in managing OA and disease progression in the long term [39].

Curcumin is extracted from the Indian spice turmeric, it is an aromatic molecule with an anti-inflammatory effect that in vitro studies showed to inhibit the activity of COX-2 and 5-LOX enzyme, thus protecting chondrocytes from the negative effects of IL-1β [57,58]. Data from literature also show in vitro a possible synergic effect of curcumin with capsaicin or resveratrol by regulating signaling of both nuclear factor-κB and IL-1β and modulating the activity of collagenase, hyaluronidase, and elastase, thus favoring the integrity of the extracellular matrix of cartilage and the differentiation and survival of chondrocytes [59]. Moreover, in vitro data show a synergistic effect of curcumin with the n-3 polyunsaturated fatty acids (PUFA)s, EPA and DHA, in enhancing antioxidant and inflammatory cytokine modulation [60]. Lastly, curcumin seems to be well tolerated at doses of 2–10 g daily, even if it should be used with caution in individuals on antiplatelet and anticoagulation therapy [39].

The oleoresin from the Boswellia serrata tree is rich in boswellic acids, which exhibit an anti-inflammatory behavior by inhibiting leukotriene synthesis by inhibiting the activity of the enzyme 5-lipoxygenase through a non-redox reaction [61]. Data from literature show and support the potential of Boswellia serrata oleoresin for the treatment of several inflammatory diseases including RA, OA, and asthma [39]. However, in relation to its anti-inflammatory mechanism, scientific data hypothesize that the higher levels of boswellic acids may modulate inflammation via prostaglandin E synthase-1 and the serine protease cathepsin G, rather than 5-lipoxygenase inhibition [62]. From randomized clinical trials, oleoresin from Boswellia serrata seems to improve the status of pain, mobility, and swelling in patients with knee OA [39,63]. However, the real effect of oleoresin from Boswellia serrata is still difficult to determine [39,64].

Phytoflavonoids and bioflavonoids are polyphenols extracted from plants and vegetables with strong anti-inflammatory and free-radical scavenging antioxidant properties [65,66,67,68]. In vitro and in vivo studies highlight that they can influence some of the metabolic and biochemical processes causing the development and progression of OA such as inhibition of the production of proinflammatory cytokines, including IL-1β, TNF-α, IL-6, and prostaglandin E2 in affected joints [69,70,71]. Moreover, in vivo, many flavonoids seem suppress inducible nitric oxide synthase, inhibiting the production of nitric oxide and neutralizing other reactive nitrogen species (RNS) and reactive oxygen species (ROS), such as superoxide [72,73] that are involved in promoting inflammatory gene expression [74]. Clinical efficacy, in OA, of phytoflavonoids and bioflavonoids is nowadays considered, in fact some authors showed improvements in WOMAC (Western Ontario and Mc Master University) OA index scores and joint function, with a low toxicity and a good safety profile [75,76].

Harpagophytum procumbens is a South African plant known as devil’s claw. In its extract, harpagosides-triterpene glycoside compounds are present which have been shown may reduce the IL-1β-induced production of matrix metalloproteinases (MMP)-1, MMP-3, and MMP-9 in chondrocytes, and downregulate TNF-α and COX-2 gene expression in vitro [77,78]. The use of devil’s claw extract as a dietary supplement in the treatment of hip and knee OA patients seems to be effective, also thanks to its safety profile and low adverse effects such as diarrhea and flatulence [79,80,81].

Bromelain is extracted from stems and immature fruits of pineapple and it contains proteolytic enzymes that may have anti-inflammatory, analgesic, antithrombotic, and antifibrinolytic properties [82]. Several clinical trials used bromelain in treatment of knee OA, with mixed and uncertain results [82,83], so that it is difficult, at this time, to give some indications in relation to its use in treatment of OA and more rigorous clinical trials are necessary.

Lastly, ginger is an anti-inflammatory and antirheumatic agent used in holistic medicine, and it contains bioactive molecules such as gingerols and shogaols [84]. In vitro, it has been shown that ginger extract suppressed TNF-α and inhibited COX-2-mediated synthesis of proinflammatory cytochines [85]. Some authors demonstrated, in clinical trials, dose-dependent improvements in the WOMAC index and visual analog scale pain profiles for knee OA, thanks to ginger extracts [86,87,88].

## 4. Conclusions

The management and treatment of OA is based on the use of anti-inflammatory agents and analgesics, surgical procedures, and rehabilitation to enable healthy body weight, lifestyle, and physical activity. However, nutritional intervention represents an ongoing strategy for managing and preventing OA as a complement to traditional clinical treatment. Nutritional interventions could regulate the balance between anabolic and catabolic processes in joint tissue, influencing immune response, redox balance, and free-radical scavenging, thus providing for structural precursors of synovial fluid and extracellular matrix of cartilage (Table 1). Many of the nutraceuticals that we describe in our review, such as GAGs and botanical extracts, show beneficial effects on joints so that nutraceutical intervention is, nowadays, considered a strategic tool for managing and preventing OA, given its risk-benefit ratio and low cost. Moreover, scientific data demonstrate that this kind of strategy supports not only clinical symptoms and functional improvement, but it could determine a regression of the disease. Although in the scientific literature there are many papers that deal with alternative treatments in the management of OA, the aim of our descriptive review is to highlight the importance of non-invasive strategies in the treatment of OA, like non-pharmacologic therapy, through the use of the most common nutraceuticals, particularly less severe forms of the disease.

## Figures and Tables

**Table 1:** Potential role of nutraceuticals in osteoarthritis. Potential role: none “−“; low “+”; moderate “++”; high “+++”.

Analyzed Articles	Nutraceuticals	Potential Role				
		Antiinflammatory	Redox Balance/Antioxidant	Anabolic	Anticatabolic	Structural Substrates
Boe and Vangsness; *Am. J. Orthop.* 2015, *44*, 302–305.	Fish Oil EPA + DHA (2–4 g/day)	+++	−	−	+++	++
Hill et al.; *Ann. Rheum. Dis.* 2016, *75*, 23–29.						
Gao et al.; *Ann. Rheum. Dis.* 2016, *75*, e13.						
Lopez; *PM R* 2012, *4*, S155–S168.						
Kirkham and Samarasinghe; *J. Orthop. Surg.* 2009, *17*, 72–76.	GAGs glucosamine sulfate (20 mg/kg body weight/day); chondroitin sulfate (1200 mg/d); hyaluronic acid (50–100 mg/d)	+	+	++	+++	+++
Michel et al.; *Arthritis Rheum.* 2005, *52*, 779–786.						
Kahan et al.; *Arthritis Rheum.* 2009, *60*, 524–533.						
Jackson et al.; *Osteoarthr. Cartil.* 2010, *19*, 297–302.						
Lopez; *PM R* 2012, *4*, S155–S168.						
Gruenwald et al.; *Adv. Ther.* 2009, *26*, 858–871.						
Maneiro et al.; *Clin. Exp. Rheumatol.* 2004, *22*, 307–312.						
Adams et al.; *Drug Saf.* 2000, *23*, 115–130.						
Balogh et al.; *J. Agric. Food Chem.* 2008, *56*, 10582–10593.						
Musumeci et al.; *J. Nutr. Biochem.* 2013, *24*, 2064–2075.	Olive oil phenolic compounds, MUFAs (500–2000 mg/d)	+++	++	+	−	+
Lopez; *PM R* 2012, *4*, S155–S168.						
Bohlooli et al.; *J. Clin. Rheumatol.* 2012, *18*, 99–101.						
Lieber and Packer; *Am. J. Clin. Nutr.* 2002, *76*, 1148S–1150S.	Methionine (800–1200 mg/d)	−	+++	+	+	++
Hosea Blewett; *Crit. Rev. Food Sci. Nutr.* 2008, *48*, 458–463.						
Kon et al.; *Knee Surg. Sports Traumatol. Arthrosc.* 2012, *20*, 436–449.						
Lopez; *PM R* 2012, *4*, S155–S168.						
Lugo et al.; *Nutr. J.* 2016, *15*, 14.	Undenatured type II collagen (40 mg/d)	+	−	+	+++	+++
Gupta et al.; *J. Anim. Physiol. Anim. Nutr.* 2012, *96*, 770–777.						
Zhu et al.; *Clin. Immunol.* 2007, *122*, 7584*.*						
Park et al.; *Mod. Rheumatol.* 2009, *19*, 581–589.						
Lopez; *PM R* 2012, *4*, S155–S168.						
Lippiello et al.; *Evid. Based Complement Alternat. Med.* 2008, *5*, 191–197.	Botanical extracts ASU (300–600 mg/d); polyphenols (300–2000 mg/d)	++	++	+	++	++
Henrotin et al.; *J. Rheumatol.* 2003, *30*, 1825–1834.						
Hong et al.; *Carcinogenesis* 2004, *25*, 1671–1679.						
Shakibaei et al.; *Biochem. Pharmacol.* 2007, *73*, 1434–1445.						
Shakibaei et al.; *Genes Nutr.* 2011, *6*, 171–179.						
Saw et al.; *Biochem. Pharmacol.* 2010, *79*, 421–430.						
-Siddiqui; *Indian J. Pharm. Sci.* 2011, *73*, 255–261.						
Abdel-Tawab et al.; *Clin. Pharmacokinet.* 2011, *50*, 349–369.						
Kimmatkar et al.; *Phytomedicine* 2003, *10*, 3–7.						
Badria et al.; *Altern. Complement. Ther.* 2002, *8*, 341–348.						
Visioli et al.; *Crit. Rev. Food Sci. Nutr.* 2011, *51*, 524546.						
Fraga and Oteiza; *Free Radic. Biol. Med.* 2011, *51*, 813–823.						
González et al.; *Crit. Rev. Food Sci. Nutr.* 2011, *51*, 331–362.						
Russo et al.; *Biochem. Pharmacol.* 2012, *83*, 6–15.						
Ahmed et al.; *J. Pharmacol. Exp. Ther.* 2004, *308*, 767–773.						
Murakami et al.; *Biofactors* 2007, *30*, 179–192.						
Ahmed; *Arthritis Res. Ther.* 2010, *12*, 208.						
Messina et al.; *Exp. Neurol.* 2009, *220*, 349–358.						
Adhikari et al.; *Indian J. Biochem. Biophys.* 2011, *48*, 275–282.						
Tseng-Crank et al.; *J. Diet. Suppl.* 2010, *7*, 253–272.						
Belcaro et al.; *Phytother. Res.* 2008, *22*, 518–523.						
Cisár et al.; *Phytother. Res.* 2008, *22*, 1087–1092.						
Schulze-Tanzil et al.; *Arzneimittelforschung* 2004, *54*, 213–220.						
Fiebich et al.; *Phytother. Res.* 2011, *10*, 36–45.						
Wegener and Lupke; *Phytother. Res.* 2003, *17*, 1165–1172.						
Gagnier et al.; *BMC Complement. Altern. Med.* 2004, *4*, 13–23.						
Brien et al.; *Evid. Based Complement. Altern. Med.* 2004, *1*, 251–257.						
Tilwe et al.; *J. Assoc. Physicians. India* 2001, *49*, 617–621.						
Semwal et al.; *Phytochemistry* 2015, *117*, 554–568.						
Frondoza et al.; *In Vitro Cell Dev. Biol. Anim.* 2004, *40*, 95–101.						
Altman and Marcussen; *Arthritis. Rheum.* 2001, *44*, 2531–2538.						

## Abbreviations

OA	Osteoarthritis
MRI	Magnetic resonance imaging
ECM	Extracellular matrix
MMP	Matrix metalloproteinases
ROS	Reactive oxygen species
TGF-β	Transforming growth factor β
GAGs	Glycosaminoglycan glycans
EPA	Eicosapentaenoic acid
DHA	Docosahexaenoic acid
HA	Hyaluronic acid
SAMe	*S*-adenosylmethionine
MUFA	Monounsaturated fatty acid
NSAIDs	Nonsteroidal anti-inflammatory drugs
UC-II	Undenatured type II collagen
ASU	Avocado/soy unsaponifiable
PUFA	Polyunsaturated fatty acid
RNS	Reactive nitrogen species
